# The influence of auditory selective attention on linguistic outcomes in deaf and hard of hearing children with cochlear implants

**DOI:** 10.1007/s00405-022-07463-y

**Published:** 2022-07-13

**Authors:** Maria Nicastri, Ilaria Giallini, Bianca Maria Serena Inguscio, Rosaria Turchetta, Letizia Guerzoni, Domenico Cuda, Ginevra Portanova, Giovanni Ruoppolo, Hilal Dincer D’Alessandro, Patrizia Mancini

**Affiliations:** 1 Department of Sense Organs, Sapienza University, Rome, Italy; 2 BrainSigns Srl, Rome, Italy; 3 Department of Otorhinolaryngology, “Guglielmo da Saliceto” Hospital, Piacenza, Italy; 4 I.R.C.C.S. San Raffaele Pisana, Via Nomentana, 401, 00162 Rome, Italy; 5 Faculty of Health Sciences, Department of Audiology, Hacettepe University, Ankara, Turkey

**Keywords:** Cochlear implants, Child, Auditory selective attention, Language skills

## Abstract

**Purpose:**

Auditory selective attention (ASA) is crucial to focus on significant auditory stimuli without being distracted by irrelevant auditory signals and plays an important role in language development. The present study aimed to investigate the unique contribution of ASA to the linguistic levels achieved by a group of cochlear implanted (CI) children.

**Methods:**

Thirty-four CI children with a median age of 10.05 years were tested using both the “Batteria per la Valutazione dell’Attenzione Uditiva e della Memoria di Lavoro Fonologica nell’età evolutiva-VAUM-ELF” to assess their ASA skills, and two Italian standardized tests to measure lexical and morphosyntactic skills. A regression analysis, including demographic and audiological variables, was conducted to assess the unique contribution of ASA to language skills.

**Results:**

The percentages of CI children with adequate ASA performances ranged from 50 to 29.4%. Bilateral CI children performed better than their monolateral peers. ASA skills contributed significantly to linguistic skills, accounting alone for the 25% of the observed variance.

**Conclusions:**

The present findings are clinically relevant as they highlight the importance to assess ASA skills as early as possible, reflecting their important role in language development. Using simple clinical tools, ASA skills could be studied at early developmental stages. This may provide additional information to outcomes from traditional auditory tests and may allow us to implement specific training programs that could positively contribute to the development of neural mechanisms of ASA and, consequently, induce improvements in language skills.

## Introduction

Selective attention represents a fundamental cognitive capacity, that allows the brain to process targeted aspects of the environment, whilst simultaneously suppressing unwanted or distracting aspects [1]. It is critical for regulating external sensory inputs that occur within and across different sensory modalities, such as vision and somatosensory processing [2].

When this ability is referred to acoustic information, it is named auditory selective attention (ASA) [3]. ASA is crucial for everyday life as we live in a noisy environment where background sounds and human voices continuously overlap, requiring us to focus on significant stimuli in a particular moment, to avoid dangers (e.g., an incoming car if we are walking along the road) or to communicate with people (e.g., when listening to our own mother who is telling us a story) without being continuously distracted by irrelevant auditory signals.

In typical development, ASA is associated with children’s lexical skills, explaining alone from 9 to 12% of variance in vocabulary scores obtained from six to seven years old children. ASA can be considered as an independent mediator in comparison to well-established factors that are significantly influencing vocabulary development (e.g., the verbal short term memory) [4]. Moreover, children with specific language impairment seem to show deficits in sustained selective attention tasks presented in the auditory modality under the high attentional load conditions, while showing similar performance to their typical developing peers on visual tasks regardless of the attentional load [5].

ASA is also highly relevant to the school setting in which instruction and completion of assignments may occur in a noisy environment with competing speech streams [6]. In situations that simulate noisy classroom settings, only children with good ASA are protected against the effects of noise in tasks where creative idea generation is required, in terms of showing performances similar to that they obtain in silence [7].

ASA depends on the ability to enhance the representation of an auditory source of interest. For this purpose, the listeners have to analyse the acoustic scene and to form a perceptual auditory object, i.e., a perceptual entity distinguished from other perceptual entities representing a stream of potentially interfering sounds [8, 9]. From this stream of sounds in an auditory scene, representing a mixture of individual sounds with various acoustic characteristics, the listeners should “segregate” the sound of interest, convey their attentional focus on it and ignore the interferers, whilst maintaining the cognitive flexibility to switch attention towards new auditory targets required by the context [9]. The entire process is complex for both adults and children. In adults, auditory object segregation and auditory attention are intertwined: the listeners need to have the ability to segregate the individual auditory stimuli that compose the complex auditory scene and to catch from it a potentially interesting sound, whilst the segregation process is biased by listeners’ desire of attention [9]. During human development, instead, segregation of concurrent stream of sounds and auditory objects formation are the primary skills that allow infants to organize the auditory input around them, “thus enabling the development of cognitive abilities such as selective attention, speech perception (distinguishing speech from nonspeech sounds and separating concurrent streams of speech from each other), social skills, and memory (by distinguishing and, subsequently, correctly representing objects)” [10].

Both the segregation of concurrent stream of sounds and the appropriate formation of an auditory object depend on proper characteristics of the auditory signals, such as intensity, temporal/spectral structure, the onset/offset time, spatial cues and timbre features [11], as well as on subjective skills in processing binaural auditory information, such as summation, squelch, and head shadow effects [12]. Consequently, deaf and hard of hearing (DHH) subjects may show limited ability in auditory object formation due to the type, severity, and symmetry of the hearing deficit. This fact may variously affect their ability to detect acoustic signals and to perform a fine analysis of their temporal and spectral cues [13]. In turn, this may make it harder for them to perceptually segregate single components of the auditory scene [14]. The distorted formation of the auditory object negatively impacts the comparison and the differentiation of objects and consequently reduces the ability to suppress irrelevant ones [8].

In quiet environments, modern hearing devices can effectively overcome perceptual limitations resulted from degradation of processing in the peripheral auditory system [15]. In noise, instead, DHH subjects, face more challenging contexts that require an increased cognitive load to fill the perceptive gaps for processing acoustic information. The cognitive load is relative both to the use of top-down strategies to select the correct auditory object and to fill in continuously the gaps left by inaudible parts in the acoustic streams of information [8].

Studies on DHH adult populations show that subjects using hearing aids or cochlear implants (CIs) experience such difficulties [16, 17]. In particular, adult DHH CI users need to base the analysis of the auditory scene on the degree of perceptual differences between the stream, owing the limited spectral and temporal resolution of CI processing [15]. Limited signal processing negatively affects their ability to benefit from bilateral CI cues and acoustic segregation as well [18]. This leads to the formation of a less robust auditory object and may explain the difficulties that CI users still face in understanding speech in more challenging listening environments with multiple speakers, background noise and reverberation [19].

Limited dichotic auditory attention performance has been reported in bilaterally implanted DHH children [20, 21], with performance comparable to that of adult DHH CI users [21]. In fact, DHH children with bilateral CIs show a limited amount of unmasking when performing the dichotic test, characterised by the ability to ignore an interferer when presented to the ear opposite to the target and by binaural unmasking when the interferer is presented to both ears [21]. According Misurelli et al. [21], these limitations might be caused by the poor peripheral encoding of speech signals that affect synchronous fusion of auditory images and central representation of the interferer.

No study up to now has investigated the influence of these limited ASA skills on language development in congenitally DHH CI children. In this context, the aim of the present study was to investigate and determine the unique contribution of ASA on the linguistic levels reached by a group of congenitally and profoundly deaf children of school age. Here, the effects of ASA were studied in respect to other personal and audiological variables that were traditionally considered to influence postoperative CI outcomes, e.g., nonverbal intelligence quotient (NVQI), age at diagnosis/implantation, family economic income (EI), maternal level of education (MLE) and auditory skills.

Differences in ASA may represent a further factor that may explain the high variability in linguistic outcomes after cochlear implantation in DHH children and this aspect needs to be investigated.

## Materials and methods

The present research was a cross-sectional study, based on the rules of the STrengthening the Reporting of OBservational studies in Epidemiology (STROBE) statement (https://www.strobe-statement.org/, last accessed 10/02/2022). The protocol was approved by the local ethics committees of the two Cochlear Implant Centers that cooperated for the study’s implementation and realization (Policlinico Umberto I Hospital, Roma; Guglielmo da Saliceto Hospital, Piacenza). The recruited families gave written informed consent for their own child’s assessment before commencing any study-related procedure.

### Participants

Thirty-four DHH CI children (21 females, 13 males) with a median age of 10.05 years (range 8–13.5 years) were included. They came from different Regions of Italy (North, Centre, and South) and were enrolled in two Cochlear Implant Centers. Table 1 showed their main demographic and clinical characteristics.

**Table 1:** Demographic and clinical characteristics of the study population (*n* = 34)

Variables	Median	Range
Age at assessment (years)	10.05	8–13.5
Age at diagnosis (months)	11.50	2–60
Age at CI (months)	18.5	7–66
Pre-CI PTA (dB HL)	101	93–110
Post-CI PTA (dB HL)	32	15–35
CPM normal score (percentile)	80	37–97

	*n* (%)
Gender	
Male	13 (38.2)
Female	21 (61.8)
Listening mode	
Monoaural CI	19 (55.9)
Bilateral CI	15 (44.1)
EI level	
Low	6 (17.7)
Middle–low	5 (14.7)
Middle	11 (32.3)
Middle–high	4 (11.8)
High	8 (23.5)
MLE	
Low (8 years)	5 (14.7)
Middle (13 years)	15 (44.1)
High (18 years)	14 (41.2)

All children had bilateral congenital profound sensorineural hearing loss, with a median preoperative pure tone average (PTA) of 101.5 dB HL (range 93–110 dB HL). Etiology of their hearing loss was as follows: unknown (*n* = 15), Connexin 26 mutation (*n* = 17), ototoxicity (*n* = 1) cytomegalovirus infection (*n* = 1). The median chronological age at diagnosis was 11.5 months (range 2–60 months), while the median age at implantation was 18.5 months (range 7–66 months). The median duration of device use at the time of assessment was 8.7 years (range 6–12.8 years).

Seventeen recipients were implanted with Cochlear devices that were fitted with ACE strategy, whilst 17 participants received Advanced Bionics devices and used Hi-Res 120 strategy. Eighteen children were bilateral CI recipients (9 simultaneous versus 9 sequential implantation), while 16 were monolateral CI users.

All CI recipients had normal cochlear conformation, with full insertion of electrode array. The absence of Central Auditory Processing Disorders and normal NVQI were verified using the Raven’s Colored Progressive Matrices-CMP [22] for children up to 11 years of age and the Raven's Progressive Matrices-RPM [20] for children between 12 and 13 years of age. The sample’s median normalized score at CPM and CMP was 80 (range 37–97).

Finally, all DHH CI children lived in monolingual native Italian-speaking environment, participated in oral rehabilitation programs, used auditory–verbal communication and were included in mainstream schools with a support teacher provided by the normal legislative procedure of Italian Ministry of Education.

Information concerning family economic income (EI) and maternal level of education (MLE) were gathered from their parents. EI was defined on the base of Italian economic family status indicator named as ISEE index (Indicatore della Situazione Economica Equivalente: Equivalent Economic Situation Index). The ISEE index based the allocation in the EI brackets computing the annual income, the real estate asset, the number of family members and the city of residence (https://www.inps.it/nuovoportaleinps/default.aspx?itemdir=50088#h3heading3). Based on this index, 5 EI brackets were defined: low, middle-low, middle, middle-high, and high. MLE was defined based on the years of formal education in three levels: low (8 years junior secondary school diploma), middle (13 years, senior secondary school diploma) and high (18 years, University degree). EI and MLE are detailed reported in Table 1.

### Assessment

#### ASA assessment

ASA skills were assessed using the “Batteria per la Valutazione dell’Attenzione Uditiva e della Memoria di Lavoro Fonologica nell’età evolutiva-VAUM-ELF” [23]. Four dichotic listening tasks, that differed each other for the weight of the distraction’s factor and for the level of cognitive workload, were used. For the distraction’s factor, there was a condition with a medium linguistic interferer (the dichotic message was a piece of television News-N, less attractive for children) and another with a high linguistic interferer (the dichotic message was a Tale-T, more attractive for children). Regarding the cognitive workload, there were two consecutive conditions: an easier condition (ASA1) with a fixed target (the word “cane: dog)” and a more difficult condition (ASA2), where the target was a semantic category, specifically the “name of an animal”. The four tasks that derived by the combination of linguistic interference and the cognitive workload conditions were: the fixed target CANE with the piece of television News as competitive message (ASA1-N); the fixed target CANE with the Tale as competitive message (ASA1-T); the target “name of an animal” with the piece of television News (ASA2-N) as competitive message; the target “name of an animal” with Tale as competitive message (ASA2-T). The difficulty of the task progressively increased from ASA1-N to ASA2-T. For every task, lists of bisyllabic words (8 target stimuli and 19 distractors) were used. The target stimuli were presented only once, while the distractors were repeated twice in random order, for a total of 46 words in each list. The duration of each test condition was 1 min and 15 s. The participant was requested to listen to the list and to raise the right hand when the target stimulus was presented, ignoring all the other words.

The tests were performed in a double-walled sound-treated booth, in sound field modality. The lists and the distractive messages were recorded and presented at the same level (65 dB SPL) through two loudspeakers positioned at 45° azimuth from the subject’s head at a distance of 1 m- one loudspeaker for the distractive message and the other one for the target message. The lists containing the target were presented to the dominant ear: to the CI side in monolateral users and to the side with the best listening performance in bilateral or bimodal CI users.

The score was calculated on number of total errors (omissions or wrong target). Normal hearing children were shown to be able to perform ≤ 1 error at ≥ 8 years of age [23]. So, after this age, a score of 2 or more errors was indicative of selective attention difficulties.

#### Auditory skills assessment

Speech recognition in quiet was assessed by using standard phonetically balanced bisyllabic words for Italian pediatric population [24]. A 10-item test list was preceded by a practice list. Items were administered in a sound-proof room, via a loudspeaker placed ad 1 m distance from a table where the child was sitting next to a speech therapist. Speech stimuli were presented at 0° azimuth at 65 dB SPL, both in quiet and with speech noise fixed at + 5 Signal-to-Noise (*S*/*N*) ratio. The participant’s score was calculated as the percentage of correctly repeated words.

The Categories of Auditory Performance-2 (CAP-2) was used to evaluate pediatric CI recipients’ auditory outcomes in daily life. This tool has been a reliable measure of outcome, with a good inter-user reliability (correlation coefficient > 0.75) [25, 26]. The CAP-2 scale consisted of 9 categories in order of increasing difficulty:

- 0. No awareness of environmental sounds.
- 1. Awareness of environmental sounds.
- 2. Responds to speech sounds.
- 3. Identification of environmental sounds.
- 4. Discrimination of some speech sounds without lipreading.
- 5. Understanding of common phrases without lipreading.
- 6. Understanding of conversation without lipreading.
- 7. Use of phone with known listener.
- 8. Follows group conversation in a reverberant room or where there is some interfering noise, such as a classroom or restaurant.
- 9. Use of phone with unknown speaker in unpredictable context.

#### Language skills assessment

The DHH CI children were tested individually in a quiet room, by two female speech therapists. All children communicated verbally, so all tests were performed using spoken language.

TwoItalian Standardized Language tests were administered to assess lexical and morphosyntactic domains. Lexical comprehension was evaluated with the Italian version of Peabody Picture Vocabulary Test (PPVT), where normal standardized scores ranged from 85 to 115 [27]. Morphosyntactic comprehension was assessed with the Italian version of the Test for Reception of Grammar (TROG)-2 [28]. Based on its standard normative data, a score < 1 SD from the mean was considered as pathologic and this was indicated in the test’s manual as the percentile ≤ 16°.

 Italian version of PPVT [27] was an assessment tool that measured the receptive vocabulary in children. It consisted of 175 black and white stimulus items, displaying 4 pictures per page with increasing difficulty. The examiner said a word, and then the examinee responded by pointing out to the picture that s/he thought to correspond to the word presented by the examiner. The raw score was calculated by subtracting the number of errors from the highest number in the examinee's ceiling set. Test–retest reliability and internal consistency of the test were 0.93 and 0.94, respectively.

Italian version of TROG-2 [28] was a fully revised and re-standardized version of the widely used TROG, originally developed to investigate morpho-syntactic comprehension skills in children. The TROG-2 consisted of 20 blocks, each testing a specific grammatical construction, having an increasing order of difficulty. Each block contained four test items and the child needed to respond correctly to all of them to level up. Each test stimulus was presented in a four-picture, multiple-choice format with lexical and grammatical foils. For each item, the examiner read a sentence that referred to one of four drawings, and the participant’s task was to point out to the drawing that corresponded to the meaning of the sentence. The score was calculated as total number of achieved blocks. Split-half reliability and internal consistency of the test were of 0.88 and 0.90, respectively.

### Statistical analysis

Analyses were carried out using a PC version of Statistical Package for Social Sciences 25.0 (SPSS, Chicago, IL, USA). Sample characteristics were reported as average and standard deviation or median and minimum–maximum values, following the analysis of normality. DHH CI children’s outcomes were compared with scale norms from the test batteries (obtained from nationally representative samples with typically developing, normally hearing children). The percentage of children performing within the normal range in the ASA tasks was reported. Wilcoxon’s test was used to assess if there were statistically significant differences between ASA performances based on the degree of the task complexity (medium vs high linguistic interferers to competitive message and low vs high cognitive workload).

The Spearman’s rank correlation coefficient was calculated to investigate the relations between the scores at the language and ASA tests, demographic characteristics (chronological age, NVQI), and audiological variables (age at diagnosis, age at implantation, duration of CI use, mono or bilateral listening, bisyllabic words recognition in quiet and in noise, CAP). Mann–Whitney and Kruskal–Wallis tests were performed to assess differences between gender, listening mode (mono and bilateral users) and mother level of education and economic income degree subgroups.

All variables with *p* values less than 0.05 in either direction were considered as significant and were afterwards used in a stepwise hierarchical multiple regression [29] to determine their contribution in predicting linguistic skills. The contribution was assessed in stages, allowing the systematic removal of different sources of information as well as the identification of the unique proportions of variance in the outcomes that could be identified with particular predictors. Variables entered on later stages are thus tested for their unique contribution after removing the contributions of earlier-entered variables.

## Results

### ASA skills

Median values and range of errors at the ASA tasks were reported in Table 2. The percentages of CI children with adequate performance were 50% in ASA1-N, 52.9% in ASA1-T, 38.2% in ASA2-N and 29.4% in ASA2-T. Both omission errors (61.4%) and wrong target errors (38.6%) were observed and their difference was statistically significant (*Z* = − 4.9, *p* < 0.001, *η*^2^ = 0.706; Cohen’s *d* = 3.101).

**Table 2:** Auditory selective attention skills of the study group (*n* = 34)

Test	Median	Range
ASA1-N (n. errors)	1	0–7
ASA1-T (n. errors)	1.5	0–8
ASA2-N (n. errors)	2.5	0–8
ASA2-T (n. errors)	3	0–8

No statistically significant differences were found in CI children’s responses when comparing tasks with medium and high linguistic interferers (ASA1-T vs ASA1-N: *Z* = − 1.14; *p* = 0.25; ASA2-T vs ASA2-N: *Z* = − 1.32; *p* = 0.18).

Performances differed significantly for cognitive workload: the number of errors from the tasks with semantic category was significantly higher than those from the task with the fixed target (ASA1-T vs ASA2-T: *Z* = − 3.15, *p* = 0.002, *η*^2^ = 0.292; Cohen’s *d* = 1.284; ASA1-N vs ASA2-N: *Z* = − 3.59, *p* < 0.001, *η*^2^ = 0.379; Cohen’s *d* = 1.563).

Bilateral CI children performed better than monolateral CI children (*U* value SA1-N = 79, *p* = 0.021, *η*^2^ = 0.143; Cohen’s *d* = 0.816; *U* value SA1-T = 73, *p* = 0.013, *η*^2^ = 0.171; Cohen’s *d* = 0.908; *U* value SA2-N = 73.5, *p* = 0.014, *η*^2^ = 0.168; Cohen’s *d* = 0.9; *U* value SA2-T = 76.5, *p* = 0.018, *η*^2^ = 0.154; Cohen’s *d* = 0.854).

### Listening and linguistic skills

Detailed scores for listening and linguistic skills were reported in Table 3.

**Table 3:** Listening and language outcomes of the study population (*n* = 34)

	Median	Range
Word recognition in quiet (%)	100	60–100
Word recognition at + 5 *S*/*N* ratio (%)	80	10–100
CAP-2 (n. of category)	7	4–8
PPVT (normal standardized scores)	90	55–125
Trog-2 (percentile)	30°	1°–90°

Median bisyllabic words recognition percentages were 100% (range 60–100%) in quiet and 80% (range 10–100%) in the presence of speech noise at + 5 *S*/*N* ratio. Thirty children (88%) showed very high auditory performances (CAP ≥ 7), reflecting the ability to communicate in more complex situations, such as noisy or reverberant environments or conversation at phone. The remaining four children (12%) showed a need to stay in a quiet setting; despite being poorer performers, they had anyway the ability to understand language without lipreading (CAP 4–6).

Standard Peabody median score was 90 (range 55–125) with 64.7% of children falling within the normal range for lexical comprehension. The median standard score at TROG-2 was 30° percentile (range 1°–90°) with 67.6% of children achieving normal scores for morpho-syntactic comprehension.

### Relationships between ASA and language skills

ASA findings correlated significantly with all language tests (Table 4). The strength of their correlations with both lexical and morphosyntactic comprehensions were moderate. Likewise, all ASA subtests were strongly correlated to each other (all Rho scores > 0.8, *p* < 0.001) and the same was true for the test of language assessment (all Rho scores > 0.75, *p* < 0.001).

**Table 4:** Spearman’s correlations between auditory selective attention tasks and language skills

	ASA1-N		ASA1-T		ASA2-N		ASA2-T	
	Rho	*p*	Rho	*p*	Rho	*p*	Rho	*p*
Peabody	− 0.51	**0.002**	− 0.49	**0.004**	− 0.46	**0.006**	− 0.49	**0.003**
TROG-2	− 0.51	**0.002**	− 0.55	**0.001**	− 0.40	**0.018**	− 0.47	**0.005**

Statistically significant values were set at *p* < 0.05 and are highlighted in bold

Owing these statistically significant correlations, a principal component analysis (PCA) was adopted to reduce the number of variables for further analysis [30]. The purpose of PCA was to derive weighted linear combinations of the individual measures that were strongly correlated, thus reducing redundancy in multiple regression analysis where the principal components were used as outcome variables. Components were more robust and representative of the study domain than any single test measure. Two new categories were identified: the linguistic component-LC (Peabody, TROG-2) and the ASA component-ASAC (ASA1-N/T, ASA2-N/T). Their Principal Components Loadings were shown in Table 5. In both analyses, PCA gave rise to one single component with a good efficiency, as indicated by the Kaiser–Meyer–Olkin (KMO) values (0.71 for LC and 0.743 for ASAC) and Bartlett p values (< 0.001 for both the components).

**Table 5:** Principal components loadings for auditory selective attention (ASAC) and linguistic (LC) components

Components	Loadings
Linguistic (LC)	
Peabody	0.94
TROG-2	0.94
Total variance explained 88.3%	
Auditory selective attention (ASAC)	
SA fixed target/tale	0.95
SA semantic target/tale	0.94
SA fixed target/news	0.93
SA semantic target/news	0.96
Total variance explained 89.9%	

A new bivariate analysis was then performed to evaluate the correlations between the new components, and the results were still statistically significant (Rho = − 0.696, *p* < 0.001).

### The unique contribution of ASA on language skills

A regression analysis was conducted to assess the unique contribution of ASA on language skills.

LC was used as the dependent variable and ASAC as the independent variable. For identification of other variables to be included in the regression analysis, significant factors in influencing LC were identified using Spearman correlation, Mann–Whitney, or Kruskal–Wallis tests depending on the nature of the variables.

Spearman’s correlation test showed statistically significant effects of NVQI assessed by CPM as well as of age at diagnosis/implantation and listening skills (Table 6).

**Table 6:** Spearman’s correlations between linguistic component and demographic and audiological quantitative variables

	Linguistic component	
	Rho	*p*
Age at assessment	− 0.20	0.25
NVQI	0.47	**0.005**
Age at diagnosis	− 0.51	**0.002**
Age at CI	− 0.42	**0.013**
Duration of CI use	0.073	0.68
Post-CI PTA	− 0.08	0.96
CAP-2	0.59	**< 0.001**
Speech in quite	0.43	**0.01**
Speech noise	0.52	**0.001**

Statistically significant values were set at *p* < 0.05 and are highlighted in bold

The Mann–Whitney test did not reveal any statistically significant differences in language performances neither because of gender (*U* = 110.5, *p* = 0.347), nor because of mono/bilateral listening mode (*U* = 110, *p* = 0.241). Mother’s degree of education was analyzed depending on the achievement of a junior, high secondary school or university degree. The Kruskal–Wallis test showed that children from families with mothers of senior secondary school or university degree (13 and 18 years, respectively) had better linguistic skills than those with junior secondary school diploma (*H* = 14.6, p = 0.001 *η*^2^ = 0.389; Cohen’s *d* = 1.596).

ASAC and all these significant variables were added in the regression model as independent factors, using the stepwise method (Table 7). At the first step, only significantly effective demographic data, mother’s level of education-MLE, and the children’s characteristics (NVQI, age at diagnosis and at CI) were included. The only significant predictors were NVQI and age at diagnosis, which explained the 46% of variances. The earlier was the diagnosis and the higher was the intelligence quotient of the child, the better were the linguistic outcomes after cochlear implantation. At the second step, the speech perception in quiet/noise and CAP scores were included in the model. These variables together accounted for 8% of an additional variance in CI children’s language competencies. The MLE and CAP scores were significant predictors: children with higher CAP scores and with mothers of a longer educational pathway, obtained the highest scores at language tests. Finally, at the third step of the model, the ASAC was added into the model in order to measure its unique contribution. This accounted for 25% of an additional variance and together with the other significant predictors–performance intelligence quotient, speech in noise and CAP–reached the 79% of the observed variances in linguistic skills of CI children.

**Table 7:** Hierarchical regression analysis to establish the contribute of ASAC on LC

Variables	STEP 1	STEP 2	STEP 3
	*β* (*p*)	*β* (*p*)	*β* (*p*)
Maternal level education	0.24 (0.13)	**0.3 (0.04)**	0.19 (0.06)
NVQI	**0.36 (0.02)**	0.23 (0.11)	**0.37 (0.007)**
Age at diagnosis	**− 0.43 (0.007)**	− 0.17 (0.3)	0.03 (0.8)
Age at CI	− 0.21 (0.51)	0.41 (0.8)	0.02 (0.9)
Speech in quite		− 0.03 (0.8)	0.1 (0.36)
Speech in noise		0.3 (0.9)	**− 0.59 (0.002)**
CAP-2		**0.55 (< 0.001)**	**0.67 (< 0.001)**
ASAC			**− 0.67 (< 0.001)**
Δ*R*^2^		0.10	0.20
*R*^2^	0.46	0.54	0.79

Statistically significant values were set at *p* < 0.05 and are highlighted in bold

## Discussion

ASA is critical for learning and development during childhood. From the first day of birth, children receive spoken language in a complex listening environment where background noise is always present and may impair their ability to learn from the linguistic input, either by limiting the available resources for learning, or by making listening particularly straining [31]. Furthermore, background noise may distract children by leading to attentional shifts and information encoding failures, even with readily perceptible targets. Children struggle to learn words in background noise, particularly when the background noise consists of nontarget speech [32]. Noise has detrimental effects also on school achievements, since in school settings, the need to pay attention and to follow instructions or assignments that may occur in the presence of competing speech streams is essential [33]. The hearing children that are more skilled in processing the target stimuli while suppressing the information from other concurrent stimuli develop better verbal working memory, lexical and academic skills [4, 6, 7].

DHH postlingual adults and children with CIs show impaired ASA due to the CI’s limited spectral resolution [17, 20, 21]. The present study confirms poor ASA skills in a DHH CI paediatric population with worst outcomes in monolateral CI users. Only 1 out of 2 DHH CI children achieves adequate performance for the less demanding ASA task (fixed target with medium linguistic interferer) and about 1 out of 3 performs within the normal range for the more complex task (changing target and high linguistic interferer), despite most of the children of the sample show good speech perception skills (CAP-2 ≥ 7). Most of the errors are represented by omission of the target and this fact allows us to speculate that the degree of perceptual discrimination between the target and the competitive message could be at the basis of the difficulty. Owing to the limited spectral resolution, DHH CI children may sometimes fail in forming the perceptual auditory object when the perceptual acoustic similarity between the target and the dichotic message makes the entire auditory target or it’s portions imperceptible.

The present bilateral DHH CI children perform better than their monolateral peers on ASA tasks, similarly with the studies by Gordon et al. [34] and Misurelli et al. [21]. Having a bilateral CI helps DHH CI children to achieve their best performance in spatial hearing and in masking release, probably because of the availability of interaural level and timing cues, that are missing in monolateral listening condition [21, 34]. This in turn, despite high variability in the amount of release from masking between bilateral subjects [35], may condition language acquisition, since DHH children with bilateral CIs achieve significantly better vocabulary outcomes and significantly higher linguistic scores in comparison to monolateral users [36].

Regarding the contribution of ASA to linguistic skills, similarly with findings in children with typical development by Majerus et al. [4], ASA represents an independent contributor to oral language skills development in this sample of DHH CI children. ASA accounts for a 25% of variance to oral language outcomes in addition to the factors, such as cognitive level, maternal education level, early intervention, listening mode and speech perception skills that are traditionally considered when studying postoperative outcomes in paediatric CI users [37, 38]. As in hearing children, ASA seems to be implicated in language processing of DHH CI children. When children interact with other people and listen to spoken language, speech represents a complex acoustic signal, with rapidly changing stream of information having few objective boundaries. From this continuous stream of auditory input, then, children face the challenge of parsing word boundaries and extracting meaning. Furthermore, many speech sounds are discriminated mainly by subtle spectral or temporal differences on the order of tens of milliseconds and many morphemes have low perceptual salience in the context of continuous speech stream. Furthermore, the presence of environmental noise and distracting speech sounds complicate the perceptual task. Hence, it is reasonable that the ability to direct selectively the attention on a target message while ignoring and suppressing distracting information could help children to process language in a more facilitated way and this, in turn, could support them to develop better linguistic skills.

This research aims to be a first attempt in determining the impact of ASA on linguistic skills attained by DHH CI children but has several limitations due to the absence of prospective follow up, the small study sample size and the absence of tasks aiming to understand the cognitive and psychoacoustic processes that may explain the nature of its findings and the specific mechanisms of ASA in paediatric DHH CI population. For example, the development of the four components of attention, represented by arousal, orientation, allocation, and maintenance, have been studied in hearing populations and might be investigated in deaf children with CI as well [39]. Also, the use of purposely developed tasks, together with the event-related brain potential technique, may allow to examine the spectral and temporal dynamics of selective attention as observed in young typical developing hearing children by Astheimer and Sanders [40] or in hearing children with specific language impairment by Stevens et al. [41] and may give us new insights in how ASA works in DHH CI children.

Nevertheless, the present findings are clinically relevant as they highlight the importance to assess ASA skills as early as possible, reflecting their important role in language development. With simple clinical instruments, as in the present study, ASA skills could be studied at early developmental stages, even in children as young as 3 years [42]. This may provide us additional information to findings from traditional auditory tests and may allow us to gain insight into early implementation of specific training programs, that could induce improvements on standardized measures of language and contribute positively to the development of neural mechanisms of ASA [43]. In English-speaking children with specific language impairment, it has been observed that they may have difficulties with linguistic forms that are perceptually less salient, such as the past tense–ed inflection, possessive s or articles [44] and that improvements in the neural mechanisms of selective attention may facilitate perception and processing of these more vulnerable linguistics forms [43]. The early detection of ASA difficulties and the development of specific programs to train auditory attention in DHH CI children may represent a new challenge for clinicians in finding new tools for improving outcomes after cochlear implantation.

Finally, these findings suggest that even at the early postoperative phases, it is of the utmost importance to support DHH CI children with the most appropriate technology such as assistive listening devices [45, 46] or adaptive microphone systems [47] in order to improve S/N ratio in challenging listening environments, and to study the long-term effects on linguistic and academic skills.
